# Distorted Temporal Consciousness and Preserved Knowing Consciousness in Confabulation: A Case Study

**DOI:** 10.3233/BEN-2011-0341

**Published:** 2011-11-07

**Authors:** Valentina La Corte, Nathalie George, Pascale Pradat-Diehl, Gianfranco Dalla Barba

**Affiliations:** ^1^Université Pierre et Marie Curie-Paris6Centre de Recherche de l'Institut du Cerveau et de la Moelle épinièreUMR-S975ParisFrance; ^2^InsermU975ParisFrance; ^3^CnrsUMR 7225ParisFrance; ^4^AP-HPHôpital de La Pitié-SalpêtriéreService de médecine physique et de réadaptationParisFrance; ^5^AP-HPHôpital Henri MondorService de NeurologieCréteilFrance; ^6^Dipartimento di PsicologiaUniversità degli Studi di TriesteItaly

## Abstract

In this study we describe a patient, TA, who developed a chronic amnesic-confabulatory syndrome, following rupture of a right internal carotid siphon aneurysm.

Our aim was to elucidate as fully as possible the nature of TA’s impairment and to test the hypothesis of confabulation as reflecting a dysfunction of Temporal Consciousness, i.e. to become aware of something as part of a personal past, present or future.

TA’s confabulations were present in answers to questions tapping Temporal Consciousness, i.e. autobiographical episodicmemory, orientation in time and place, and foresight of personal future. In contrast, confabulations were not observed in answers to questions tapping Knowing Consciousness, i.e. to become aware of something as a meaning or as an element of impersonal knowledge. In fact, he had normal access to semantic knowledge, including foresight of impersonal future. TA’s brain MRI showed lesions involving the right hippocampus, parahippocampal gyrus, fornix, mammillary bodies, and thalamus. Moreover TA showed sub-cortical lesions involving the caudate and putamen nuclei bilaterally, a lesion site not commonly described in amnesic-confabulatory syndrome. We suggest that this pattern of results is better accounted for within the framework of the Memory, Consciousness and Temporality Theory and reflects a specific distortion of Temporal Consciousness.

## 1. Introduction

Confabulation is a symptom, observable in amnesic patients who are unaware of their memory deficit. It consists of actions and verbal statements that are unintentionally incongruous to the patient's history, background, present and future situation [4].

This rather infrequent disorder is classically described in Korsakoff's syndrome [2,9,34], but also in patients suffering from ruptured aneurysms of the anterior communicating artery, subarachnoid haemorrhage or encephalitis [21,33], head injury [1,5], Binswanger's encephalopathy [4], Alzheimer's disease [17] and aphasia [27]. Confabulating patients recall episodes and facts which never took place or that took place in a completely different context, but are experienced as real memories.

Several authors have distinguished between different forms of confabulation. Bonhoeffer [2] distinguished between *momentary confabulations* and *fantastic confabulations*. Momentary confabulations are typically produced in response to questions, are always plausible and have been considered to reflect a more or less intentional strategy to fill a gap in memory and to overcome

ISSN 0953-4180/11/$27.50 © 2011 – IOS Press and the authors. All rights reserved embarrassment. By contrast, fantastic confabulations are much more context-free, unprovoked, implausible statements relating to unreal events and often favoured by an euphoric mood.

More recently, Kopelman [19] distinguished between *provoked* and *spontaneous* confabulations. According to Kopelman, provoked confabulation reflects a normal, plausible response to a faulty memory, whereas spontaneous confabulation reflects the production of incoherent memories and associations, resulting from the superimposition of frontal dysfunction on an organic amnesia.

Schnider [29] extended Kopelman's classification and proposed to distinguish between four forms of confabulation: 1. *Intrusions* in memory tests, or alternatively, *provoked confabulation*; 2. *Momentary confabulations*, which describe false verbal statements in a discussion or other situation inciting a patient to make comments; 3. *Fantastic confabulation*, which have no basis in reality, are nonsensical and logically inconceivable; 4. *Behaviourally spontaneous confabulations* occurring in the context of severe amnesia and disorientation. With this term, Schnider indicates patients who not only produce verbal confabulations, but who also behave and act on their confabulations.

Following a different perspective, Dalla Barba [4] proposed that, regardless their modality of appearance (spontaneous *vs* provoked), confabulations can be distinguished according to the semantic quality of their content. Confabulation may be *semantically appropriate*, when, because of their internal semantic coherence, they are indistinguishable from true memories, unless one has access to personal information concerning the individual who confabulates. Or, they can be *semantically anomalous*, when, because of their internal semantic incoherence, they are recognizable as confabulations, although one doesn't know anything about the individual who confabulates.

More recently, Dalla Barba and co-workers [7,20], analysing the content of 476 confabulations (both spontaneous and provoked) produced by confabulating amnesics and patients with Alzheimer's disease, proposed a new taxonomy, which identifies six types of confabulations: 1. Habits, i.e. confabulations consisting of personal habits, which are considered by the patient as specific personal episodes; 2. Misplacements, i.e. confabulations consisting of true episodes and facts misplaced in time and place; 3. Memory Fabrications, i.e. plausible memories, without any recognizable link with personal or public events; 4. Memory Confusions, i.e. confusion with other personal or public events related to the target memory or confusion between family members; 5. Autoreferential Contaminations, i.e. when patients, questioned about public or historical events, refer to the event in a personal context; 6. Semantically Anomalous Confabulations, i.e. confabulations with an extremely bizarre and semantically anomalous content. In their analysis, Dalla Barba and co-workers found that Habits Confabulations and Misplacements Confabulations were the most frequently observed type of confabulation, regardless the underlying pathology. This is consistent with clinical and experimental observation, which show that confabulation often consists either of true episodes misplaced in time and place or of personal habits which are considered by the patient as specific personal episodes [7,20]. When asked what they did today or what they will be doing tomorrow, confabulating patients may reply with well-established memories from the past, however irrelevant these memories may be to their present situation.

Confabulation has traditionally been considered a disorder of memory and, in particular, a disorder of the retrieval or post retrieval stage from long-term memory [1,3,23]. There is evidence, however, that considering confabulation as a disorder confined to long-term memory is probably restrictive. In fact, confabulators, usually make errors in time and space orientation tasks [4,5,9] and also when they are required to foresight their personal future [5,8].

A number of ideas about mechanisms underlying confabulation emphasise the role of a frontal executive dysfunction. According to the strategic retrieval hypothesis [22–24], for example, confabulation is considered a deficit of retrieval and post-retrieval processes. Following a similar perspective Johnson [16] argues that confabulation is a disorder resulting from a failure of 'reality monitoring' (i.e. deciding whether a memory is a trace of something that actually happened to you, or is a memory of an imagined event). Damage to frontal/executive functions would results in an impairment of judgment processes involved in reality monitoring and so in confabulation. Fotopoulou and co-authors argue that confabulation should be interpreted within a motivational framework and they suggest that confabulations include motivated or 'wishful' content [13].

Schnider and colleagues [28–30] suggest that the primary cause of spontaneous confabulation is a confusion of ongoing reality with past events. Accordingly confabulators fail to suppress evoked memories that do not pertain to ongoing reality. Lesions typically involve the posterior medial orbitofrontal cortex or its connections in the basal forebrain [31]. Imaging and evoked potential studies in healthy subjects support the idea that the anterior limbic system subserves a reality monitoring mechanism which selects memories of current relevance, while filtering currently irrelevant memories [31].

According to Dalla Barba and co-workers [7,10], confabulation is not a pure memory disorder, but a disorder involving Temporal Consciousness. Temporal Consciousness means to become aware of something as part of a personal past, present or future. These ideas are part of Memory Consciousness and Temporality Theory, which is detailed elsewhere [6]. According to this theory, in confabulating patients Temporal Consciousness is still there, like in normal subject, therefore these patients can still remember their past, they are present to a world and can project themselves into a personal future. However, in doing this they make confabulatory errors, that are the result of mistaking habits and routines as past or future personal episodes, plans and events. In a recent study, in which we analysed confabulations' content in mild Alzheimer's disease patients and in confabulating amnesics, we found that the majority of confabulations were "Habit Confabulations", i.e. repeated personal events mistaken as unique past and future personal episodes [20]. These results indicate a distortion of Temporal Consciousness, in that Temporal Consciousness considers atemporal over learned information within a specific temporal context.

There is increasing converging evidence that lesions which produce amnesia also produce a loss of Temporal Consciousness. In fact amnesic patients not only do not consciously remember their past, but neither can they imagine their personal future [18,35]. This suggests that the integrity of the medial temporal lobe (MTL) is crucial for the function of normal and confabulatory Temporal Consciousness. In fact, the metanalysis realized by Gilboa and Moscovich [14] showed that only 2 out of 79 patients with confabulation had unilateral lesions involving the MTL. Twenty-eight additional confabulating patients not included in Gilboa's and Moscovich's had also preserved MTL [7]. Taken together these data clearly show that what confabulators have in common is not a specific lesion site, but rather the integrity of the MTL, which is consistent with the idea that the MTL is essential for the function of normal and confabulatory Temporal Consciousness.

In this paper we describe a patient, TA, who developed a chronic amnesic-confabulatory syndrome, following rupture of a right internal carotid siphon aneurysm.

Our aim was to elucidate as fully as possible the nature of TA's impairment and to test the hypothesis of confabulation as reflecting a dysfunction of Temporal Consciousness. TA suffered from a profound loss of episodic memory; by contrast his semantic memory was relatively spared. His confabulations were limited to episodic memory, orientation in time and place and personal future, thus reflecting a dysfunction of Temporal Consciousness. In addition TA showed subcortical lesions that, to our knowledge, have never been described in confabulation.

## 2. Case report

### 2.1. History

At the time of assessment, TA was a 39-year-old right-handed man. He was married and had two children. He had no history of psychiatric or neurologic diseases. He was a geologist working for an oil company and he had travelled widely for professional reasons. On January 2008 he was admitted to the hospital with a massive basal subarachnoidal hemorrhage.

### 2.2. Neuropsychological examination

TA was tested on various occasion between November and December 2008 (Table 1). His collaboration during the testing sessions was good. TA showed deep anterograde amnesia: he was unable to retain new information for more than a few seconds and on direct questioning he was disoriented and confabulated in time and space.

He performed well on certain tasks which are in general considered to be sensitive to intellectual deterioration, such as the Raven's Coloured Progressive Matrices-PM 47, where he achieved a score of 35/36. In contrast he performed poorly on the Mini Mental State Examination [12] mainly due to his disorientation in time and place.

TA was completely unaware of his memory deficits and he never admitted explicitly having memory problems, either spontaneously or when specifically questioned by the examiner. On bedside tests of oral expression, understanding of oral language, reading, writing, neglect, praxis and calculation, the patient was judged to be normal.

The evaluation of episodic memory showed a severe deficit both in the verbal and visual domains. In particular on the Free and Cued Selective Reminding

Table 1
TA'scores on the Neuropsychology assessment. MMSE = Mini-Mental State Examination, PM47 = Progressive Matrices 1947, PTT = Pyramids and Palm Tree Test, FCRT = Free and Cued Selective Reminding Test, FAB = Frontal Assessment Battery, MCST = Modified Card Sorting Test

| | TA | Comments |
|---|---|---|
| Intellectual efficiency | 18/30 | Controls' mean = 29, S.D. = 1.7 |
| MMSE | 35/36 | Centile = 90, IQ = 125 |
| PM47 | | |
| Semantic memory | 38/40 | Pathological score < 35 |
| Naming | 9 | Controls' mean = 36.8, S.D = 8.7 |
| Verbal fluency semantic | 6 | Controls' mean = 25.7, S.D = 6.3 |
| Verbal fluency phonemic | 38/40 | Pathological score < 35 |
| PTT (words) | | |
| Episodic memory | 4/36 | < 10 centile |
| Rey Figure | 2/48 | Controls' mean = 34.7, S.D = 4.9 |
| FCRT | 9/46 | Controls' mean = 11.6, S.D = 3.9 |
| Total free recall | 27 | |
| Total cued recall | | |
| Number of intrusions | | |
| Executive functions | 11 | Controls' mean = 3.6, S.D = 1.92 |
| Cognitive Estimate | 13/18 | Controls' mean = 17.3, S.D = 0.8 |
| FAB | 3/6 | Controls' mean = 5.93, S.D = 0.38 |
| MCST | 67 | Cut-off for frontal lobe lesion = 50 |
| Categories achieved | | |
| % Perseverative errors | | |

Test [15] his performance was massively contaminated by intrusions, especially in cued recall. Moreover his performance was also severely impaired on the recall of Rey's Figure.

TA's performance in tasks involving various types of semantic knowledge was normal: definition of words, performing a symbolic gesture on spoken request, naming and semantic association tasks, e.g. the Pyramids and Palm Tree Test.

As confabulation has often been in relation with lesion involving the frontal lobe structures, his performance was examined on tasks tapping executives functions. His performance was impaired both on the Cognitive Estimates [32] and on the Modified Card Sorting Test [26].

On Category Fluency, a test that require generating as many words as possible in 2 minutes from a given semantic category, e.g. animals, he produced an average of 9 words and on Letter Fluency, involving generating as many words as possible starting with letter F he produced an average of 6 words. His performance on Frontal Assessment Battery [11] was severely impaired. This battery contains subtests that are considered sensitive to frontal lobe dysfunction. TA was impaired on mental flexibility, motor programming (Luria sequencing test) and in sensitivity to interference. He performed normally on conceptualization and environmental autonomy subtests.

### 2.3. Examination of confabulation

TA produced most of his confabulations in informal conversation, when answering specific questions and spontaneously, mainly referring to autobiographical episodic memory.

Confabulation was always plausible and semantically appropriate so that a hypothetical observer not familiar with TA's history, background and present situation could not tell whether TA's reports were confabulatory or not. For example he would claim that the day before he was at university to give a lecture.

Confabulations were collected with the Confabulation Battery [(CB) [4]]. The CB involves the retrieval of various kinds of information and consist of 165 questions, 15 for each of the 11 following domains: Personal Semantic Memory, Episodic Memory, Orientation in time and place, Linguistic Semantic Memory, General Semantic Memory (Recent, Contemporary, Historical), Semantic Plans, Episodic Plans and questions to which the appropriate response would be 'I don't know', both semantic and episodic. Each domain has been described in detail elsewhere [10,20] Questions from 11 domains were administered to TA and NC in a semi-randomized order. Responses were scored as 'correct', 'wrong', 'I don't know' and 'confabulation'. For episodic memory, responses were scored 'correct' when they matched information obtained from the patient's relatives. Correct responses were selfevident for semantic memory questions. For 'I don't know' questions, both Semantic and Episodic an 'I don't know' response was scored as correct. Because there is no sufficiently acceptable external criterion capable of defining confabulation, for its detection an arbitrary decision necessarily had to be made.

To distinguish between a wrong response and a confabulation, a clear-cut decision was adopted only for answers to questions probing orientation in time. In this case the most strict criterion was chosen: answers to questions regarding the current year, season, month, day of the month, day of the week, and hour of the day were judged to be confabulations only if erring for more than 5 years, 1 season, 2 months, 10 days, 3 days or 4 hours, respectively. Minor distortions were considered errors, whereas major discrepancies between the expected and the given answer were considered confabulations, regardless of their content.

Answers concerning the future can be definitely confabulatory when they show a marked discrepancy with what a predicted future event might be, in view of the present situation.

As it is clearly emerges from Fig. 1, TA's confabulations were found to be restricted to episodic memory, orientation in time and place and in episodic plans, thus reflecting a dysfunction of Temporal Consciousness, i.e. the patient's personal past, present and future. In contrast he never confabulated on questions tapping non-personal semantic memory. Confabulation probing Temporal Consciousness was always plausible and mostly of the Habit Confabulation type, i.e. routines and atemporal repeated personal episodes [20]. For example to the question 'what did you do yesterday?' he answered 'I was at the Montpellier University to give a lecture'. Actually he had lived in Montpellier and he had worked at the University. So, the patient recalled a true autobiographical information, but this information was not coherent with the ongoing reality.

He produced Habits Confabulations also in answering questions concerning the orientation in time and place and on episodic plans. For example to the questions: 'what are you going to do tonight?' he answered 'I'll go back home and I will have dinner with my wife'. This response is based on habits and daily life routines rather than specific episodic plans. Habits Confabulations were also observed in answers to questions probing orientation in time and place. For example, to the question 'where are we now?' he answered: 'We are in the French Oil Department', a place where the patient had actually been working some years before.

### *2.4. Lesion' site*

Confabulation has often been associated to lesions involving the ventromedial portion of the frontal lobes and related structures, including the basal forebrain, septum, and anterior hypothalamus (see [29]). However, confabulations are also described for lesions not involving the frontal lobe and related structures [4,8,9].

An MRI performed in July 2008 showed that TA had a number of lesions both cortical and subcortical. In particular, he showed lesions involving the right hippocampus, the parahippocampal gyrus bilaterally, although more on the right, the thalamus, fornix and mammilary bodies. Moreover, TA showed lesions involving the caudate nuclei, more pronounced in the right, and the putamen bilaterally (Fig. 2). To our knowledge, these latter lesion sites have only been described once in confabulation [25].

## 3. Discussion

The patient we described had a florid confabulatory syndrome confined to Temporal Consciousness, i.e. conscious awareness of personal past, present and future, in the context of preserved general intellectual abilities. Indeed, the most interesting aspect of TA's case is that his confabulation affected the three dimensions of temporality – past, present and future. In others words, in this patient [but see also [5,8]] confabulation does not involve only the retrieval of personal past episodes and present orientation in time and place, but manifest itself also when he is asked to plan his personal future. The hypothesis put forward in this work was that TA suffers from a distortion of Temporal Consciousness, whereas his Knowing Consciousness is preserved. This hypothesis is confirmed by the results of the Confabulation Battery, where he shows a clear-cut dissociation between his impaired ability to answer questions concerning his personal temporality – past, present and future – and his preserved capacity to respond appropriately to questions concerning his impersonal temporality, i.e. knowledge of past and future public events.

TA confabulated both spontaneously and in answering specific questions, like in the Confabulation Battery. Therefore, the provoked/spontaneous distinction [19] does not apply to his case. TA can neither be classified as a "behaviorally spontaneous" confabulator [29] since he never acted on his confabulations. In contrast, his confabulations were always semantically ap-

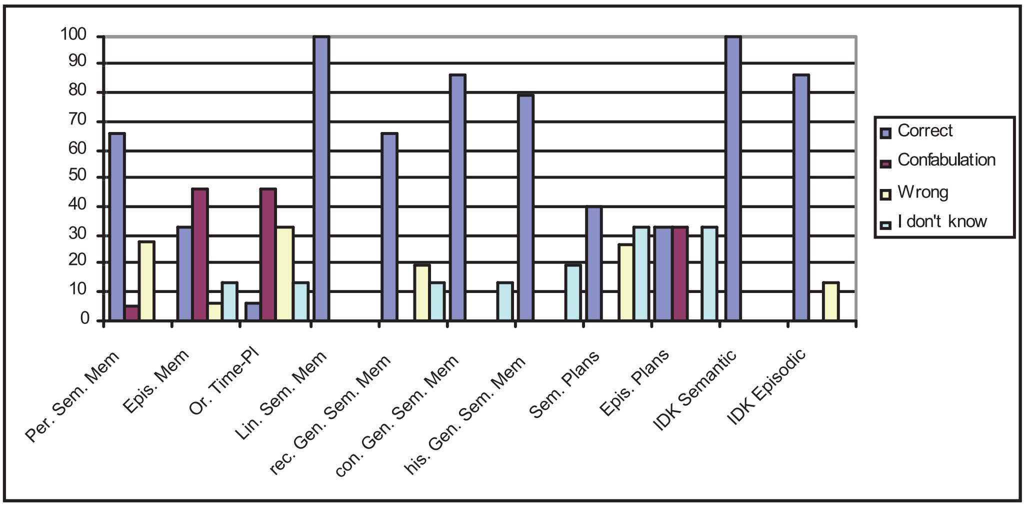

Fig. 1. TA percentages mean at different domains of Confabulation Battery.

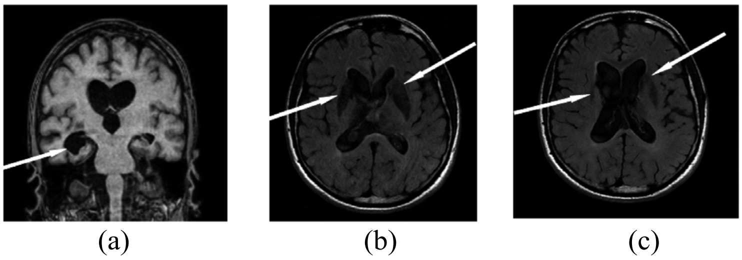

Fig. 2. a: Coronal T1-weighted brain MRI scan showing lesions involving the right hippocampus and parahippocampus; b and c: Axial FLAIR images showing right and left sub-cortical lesions with particular involvement of the caudate nuclei and putamina.

propriate [4] and invariably consisted of habits or true memories misplaced in time and place [20].

Most studies concerning the effects of amnesia on temporal experience have focused on the role of memory in reconstructing one's personal past. However, the study of personal temporality as a whole in amnesia has been largely neglected. TA's case shows that positive memory symptoms, like confabulation, involve the whole subjective temporality, leaving almost intact impersonal temporality, i.e. the access to past and future semantic information, as well as knowledge of 'physical' or chronological time. In fact, TA had normal knowledge of past public and historical events, and, although disorientated, had preserved knowledge of units of time and their relationships, i.e. he knew what an hour, a day, a week, a month and a year are.

TA's case shows several analogies with the case of patient G.A described by Dalla Barba and co-workers [8]. GA developed an amnesic-confabulatory syndrome following a subarachnoid haemorrhage and ischemia due to rupture of the anterior communicating artery and subquent vasospasm. G.A suffered a profound loss of episodic memory. By contrast her semantic memory was relatively spared. GA's confabulation involved the autobiographical aspect of episodic memory as well as the orientation in time and place and also the planning of her personal future. GA was the first reported case in the literature in which confabulations affected the three dimensions of subjective temporality. A deficit of Temporal Consciousness with preserved access to past and future semantic information has also been described in amnesic patient DB [18]. DB, as a result of hypoxic brain damage, suffered severe amnesia for the personally experienced past. By contrast, his knowledge of non personal past was relatively preserved. A similar pattern was evidenced in his ability to anticipate future events. In fact, although DB had great difficulty imagining what his experiences might be like in the future, his capacity to anticipate issues and events in the public domain was comparable to that of healthy, age-matched controls. DB also produced some confabulations, which were restricted to questions probing

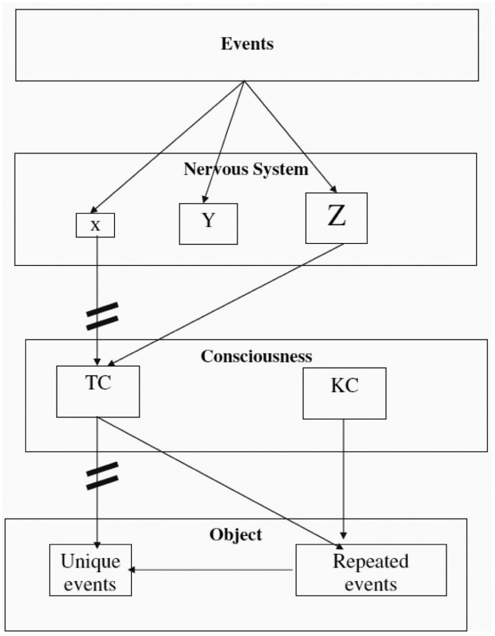

Fig. 3. Confabulation according to the Memory, Consciousness and Temporality Theory [6]. NS = nervous system; X, Y, Z = less stable (X), more stable (Z) patterns of modification of the nervous system; TC = temporal consciousness; KC = knowing consciousness. Arrows indicate the succession order of phenomena from events to consciousness in normal and confabulating memory.

personal past and personal future. In DB, like in patient GA, there was no evidence of hippocampal damage.

The pattern of confabulation showed by TA reflects the same kind the dissociation observed in GA and DB, i.e. a distorted personal temporality as opposed to preserved access to past and future impersonal information. This dissociation between distorted personal temporality and preserved impersonal temporality can be accounted for within the framework of the Memory Consciousness and Temporality Theory (MCTT). According to the MCTT, confabulation is considered as a dysfunction of Temporal Consciousness. Aspects of MCTT relevant to the interpretation of confabulation are summarized below and schematically represented in Fig. 3.

– Events produce atemporal and aspecific modifications in the brain. These modifications, represented in Fig. 3 as X, Y and Z, are atemporal in the sense that they do not contain any information concerning time. They are aspecific in the sense that they do not contain any information specifying that they are representing episodes, meanings, rules, etc.
– The modifications in the brain can be more or less stable or more or less vulnerable depending on a number of variables. These variables include, attention at encoding, emotional value of the event, depth of encoding, repeated experience of the same event etc..
– Consciousness means to be conscious *of something in a specific way*. That means that consciousness is not an aspecific dimension that passively receives different types of already specified information and become aware of it, but rather that different types of consciousness exist, each representing an original and irreducible way of addressing the world.
– Different types of consciousness include, among others, Temporal Consciousness and Knowing Consciousness. Temporal Consciousness means to become aware of something as part of a personal past, present or future, Knowing Consciousness means to become aware of something as a meaning or as an element of impersonal knowledge (i.e. information not referring to specific personal episodes).
– The object of consciousness represents a determination and an undetermination, what we have called U(niqueness) and M(ultiplicity). U refers to unique events, whereas M refers to repeated events. Temporal Consciousness addresses the object's U, whereas Knowing Consciousness addresses its M. For example, this pen on the desk is both *a pen* and *the pen.* In the first case it is an undetermined pen, something that belongs to the category of 'pens', an object that I recognize and use appropriately because I recognize it. In the second case, 'the pen' is a determined object, it is exactly this pen in front of me, the pen I bought yesterday and that I will be using tomorrow. So the pen reveals a U and a M. M is reflected in its being a pen and not a different object. U manifests itself in its being precisely this pen and not another pen.

In patients who confabulate we are faced with a confabulatory Temporal Consciousness, in which past, present and futures are somehow distorted. In confabulating patients Temporal Consciousness is present, as in normal subject. These patients can still remember their past, they are present to a world and they can project themselves into a personal future. But in doing this they make errors, sometimes frankly bizarre errors. What is actually happening in these patients is that Temporal Consciousness is present, but it is not interacting with less stable patterns of modification of the brain, because these modifications are abolished or inaccessible. Most of the time the result of this condition is that repeated events, personal habits and routines are considered in a personal temporal framework. When asked what they have done the previous day or what they are going to do the following day, confabulating patients typically answer with memories and plans that they usually have in their daily life. Although admitted to the hospital, they will say, for example, that they went out shopping the previous day and that they will be visiting some friends the following day, acts that presumably were part of their routine life. According to the MCTT, in this condition, Temporal Consciousness interacts with more stable patterns of modification of the brain and addresses the object's M – habits, routines, repeated events, as U, as specific unique past events (Fig. 3).

Conversely, in non confabulating amnesic patients we observe a loss of Temporal Consciousness. These patients not only do not consciously remember their past, but neither can they imagine their personal future. They are lost in a *non time*, a sort of instantaneous present. A good description of this condition comes from observations of patient K.C., who, as a result of a severe brain injury was severely amnesic. When informally interviewed on his plans for the future, K.C, was unable to imagine anything he was likely to do on any subsequent occasion [35].

It has been argued that disorientation is typical of some subtypes of confabulation, whereas it would be loosely associated to other subtypes of confabulation and to non confabulatory amnesia (see [29, pp. 151-157] for a discussion of this topic). It is well known that disorientation may have diverse mechanisms, tend to vary from one testing session to the next, and, above all, it varies according to how it is measured. However, regardless the underlying pathology, clinically "positive", i.e. productive disorientation is invariably present in confabulation. On the other hand, clinically, nobody would describe non confabulating amnesics as people normally oriented in their present world. Actually, disorientation is one aspect of distortion, in confabulators, or loss, in non confabulating amnesics, of Temporal Consciousness.

According to the 'temporal context confusion theory' [28,29] confabulation results from the inability to suppress activated memory traces that do not pertain to ongoing reality. This account also puts the role of temporality as crucial in confabulation. According to this view TA confabulation reflects the inability to suppress memories and autobiographical events that are not pertinent with the present situation. This view is compatible with our account, which further specifies that unsuppressed memory traces represent habits or repeated events – the object's Multiplicity in the MCTT.

Within the framework of strategic retrieval account, [23] the disruption of the monitoring subcomponent of strategic retrieval would be responsible for confabulation. However there are some difficulties in considering TA's confabulation as the result of a monitoring deficit. If this were the case confabulation would have appeared every time the strategic component of retrieval was involved, regardless the type of retrieval semantic or episodic. By contrast TA confabulated only in episodic memory tasks, whereas in semantic memory tasks he either was correct or he responded 'I don't know', even when strategic retrieval was likely to be involved.

Our case study confirms that confabulation should be considered as a distortion of Temporal Consciousness, as a whole. TA remembers another past, lives another present and he projects himself in an unreal future. In contrast, his Knowing Consciousness is preserved, since he has normal access to semantic information, both past and future.

TA showed lesions involving the right hippocampus, parahippocampal gyrus, fornix, mammillary bodies, and thalamus. Moreover he showed sub-cortical lesions involving the caudate and putamen nuclei bilaterally. This latter lesion site has only been described once in amnesic-confabulatory syndrome. It is difficult to tell what the relative contribution of these subcortical lesions to the confabulatory syndrome would be. However, given the functional and structural connections of the caudate nucleus with the basal forebrain, it can be speculated that a lesion in this nucleus may contribute to confabulation by disabling extinction capacity, that is, failure to integrate negative prediction errors into behavior [25].

TA had preserved left hippocampus. Unlike classic amnesic patients, confabulating amnesics have preserved hippocampus, at least unilaterally [14]. These patients may have lesions, either focal or diffuse, in more than twenty brain sites, and accordingly confabulation may vary in severity and, possibly, in type of content. But, rather than a specific lesion site, what seems to be crucial for the production of confabulations is the preservation of the hippocampus and of the MTL. According to our view, confabulators have a distorted Temporal Consciousness and this might reflect the disconnection, either upstream, or downstream, of a preserved MTL from other brain structures.
